# Software for the analysis and visualization of deep mutational scanning data

**DOI:** 10.1186/s12859-015-0590-4

**Published:** 2015-05-20

**Authors:** Jesse D Bloom

**Affiliations:** Division of Basic Sciences and Computational Biology Program, Fred Hutchinson Cancer Research Center, 1100 Fairview Ave N, Seattle, 98109 WA USA

**Keywords:** Deep mutational scanning, Sequence logo, Amino-acid preferences

## Abstract

**Background:**

Deep mutational scanning is a technique to estimate the impacts of mutations on a gene by using deep sequencing to count mutations in a library of variants before and after imposing a functional selection. The impacts of mutations must be inferred from changes in their counts after selection.

**Results:**

I describe a software package, dms_tools, to infer the impacts of mutations from deep mutational scanning data using a likelihood-based treatment of the mutation counts. I show that dms_tools yields more accurate inferences on simulated data than simply calculating ratios of counts pre- and post-selection. Using dms_tools, one can infer the preference of each site for each amino acid given a single selection pressure, or assess the extent to which these preferences change under different selection pressures. The preferences and their changes can be intuitively visualized with sequence-logo-style plots created using an extension to weblogo.

**Conclusions:**

dms_tools implements a statistically principled approach for the analysis and subsequent visualization of deep mutational scanning data.

## Background

**Figure 1:**
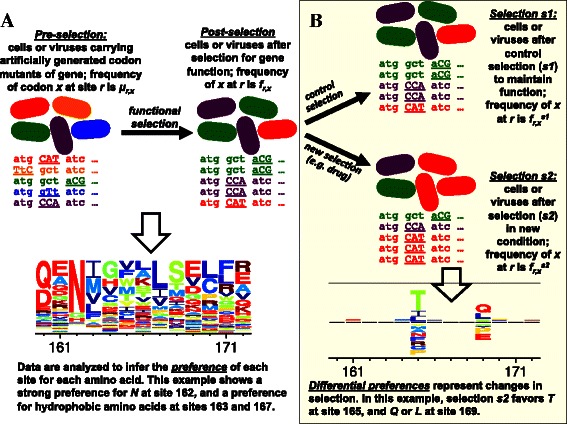
A deep mutational scanning experiment.**(A)** A gene is mutagenized to create a library that contains all single codon mutations. The mutant library is introduced into cells or viruses and subjected to a functional selection that enriches beneficial mutations and depletes deleterious ones. Deep sequencing is used to count mutations in a sample of the variants present pre- and post-selection. Using dms_tools, the data can be analyzed to infer the “preference” of each site for each amino acid; in the visualization, letter heights are proportional to the preference for that amino acid. **(B)** The experiment can be extended by subjecting the library of functional variants to two different selection pressures, and using deep sequencing to assess which variants are favored in one condition versus the other. Using dms_tools, the data can be analyzed to infer the “differential preference” of each site for each amino acid in the alternative selection *s*2 versus the control selection *s*1; in the visualization, letter heights above or below the line are proportional to the differential preference for or against that amino acid.

Deep mutational scanning is a high-throughput experimental technique to assess the impacts of mutations on a protein-coding gene [1]. Figure 1 shows a schematic of deep mutational scanning. A gene is mutagenized, and the library of resulting variants is introduced into cells or viruses, which are then subjected to an experimental selection that enriches for functional variants and depletes non-functional ones. Deep sequencing of the variants pre- and post-selection provides information about the functional impacts of mutations. Since the original description of deep mutational scanning by Fowler *et al.* [2], the technique has been applied to a wide range of genes [3-15], both to measure mutational tolerance given a single selection pressure as in Figure 1A, or to identify mutations that have different effects under alternative selections as in Figure 1B. New techniques to create comprehensive codon-mutant libraries of genes make it possible to profile all amino-acid mutations [8-10,15-17], while new techniques for targeted mutagenesis of mammalian genomes enable deep mutational scanning to be applied across the biological spectrum from viruses and bacteria to human cells [18].

A key component of deep mutational scanning is analysis of the data: First, raw reads from the deep sequencing must be processed to count mutations pre- and post-selection. Next, the biological effects of mutations must be inferred from these counts. The first task of processing the reads is idiosyncratic to the specific sequencing strategy used. But the second task of inferring mutational effects from sequencing counts is amenable to more general algorithms. However, only a few such algorithms have been described [19,20]. Here I present user-friendly software, dms_tools, that infers mutational effects from sequencing counts. Before describing the algorithms implemented in dms_tools and illustrating its use on existing and simulated data, I first discuss issues associated with inferring mutational effects from sequencing counts.

### The nature of deep mutational scanning data.

The data consist of counts of variants pre- and post-selection. The approach presented here treats each site in the gene separately, ignoring epistatic coupling among mutations. This aspect of the approach should not be construed as a suggestion that interactions among mutations are unimportant; indeed, several studies have used deep mutational scanning to examine pairwise epistasis [14,21,22], and techniques have been described to obtain linkage between distant sites [23,24]. However, the exploding combinatorics of multiple mutations (a 500-residue protein has only 19×500≈10^4^ single mutants, but $19^{2} \times \frac {500 \times 499}{2} \approx 4 \times 10^{7}$ double mutants and $19^{3} \times \frac {500!}{497! \times 3!} \approx 10^{11}$ triple mutants) make it currently plausible to comprehensively characterize only single mutations to all but the shortest genes. Treating sites independently is therefore not a major limitation for most current datasets. Eventually the approach here might be extended to include coupling among mutations.

The data for each site *r* is characterized by the sequencing *depth* (total number of counts); let $N_{r}^{\text {pre}}$, $N_{r}^{\text {post}}$, $N_{r}^{s1}$, and $N_{r}^{s2}$ denote the depth at *r* for each of the four libraries in Figure 1 (pre-selection, post-selection, selection *s*1, and selection *s*2). Typical depths for current experiments are *N*∼10^6^. Denote the counts of character *x* (characters might be nucleotides, amino acids, or codons) at *r* as $n_{r,x}^{\text {pre}}$, $n_{r,x}^{\text {post}}$, $n_{r,x}^{s1}$, and $n_{r,x}^{s2}$. The values of *n*_*r*,*x*_ for characters *x* that differ from the wildtype identity wt(*r*) depend on both the depth *N* and the average per-site mutation rate $\overline {\mu }$. Since the mutations are intentionally introduced into the mutant library by the experimentalist, in principle $\overline {\mu }$ could have any value. But typically, deep mutational scanning experiments aim to introduce about one mutation per gene to avoid filling the mutant library with highly mutated genes – so the average mutation rate is usually $\overline {\mu } \sim 1/L$ where *L* is the length of the gene. Therefore, if a 500-codon gene is sequenced at depth *N*∼10^6^, we expect $N \overline {\mu } \sim 2000$ counts of non-wildtype codons at each site. Since there are 63 mutant codons, the average pre-selection counts for a mutation to a specific *x*≠ wt(*r*) will be $n_{r,x}^{\text {pre}} \sim 30$, with counts for most mutations deviating from this average due to biases in creation of the mutant library and randomness in which molecules are sequenced. Counts in the post-selection libraries will further deviate from this average due to selection. Therefore, even at depths *N*∼10^6^, the actual counts of most mutations will be quite modest.

The rest of this paper assumes that the sequencing depth is less than the number of unique molecules in the mutant library, such that the deep sequencing randomly subsamples the set of molecules. If this assumption is false (i.e. if the number of unique molecules is substantially less than the sequencing depth), then the accuracy of inferences about mutational effects will be fundamentally limited by this aspect of the experimental design. Properly done experiments should quantify the number of unique molecules in the library so that it is obvious whether this assumption holds. In the absence of such information, the analysis can be repeated using only a random fraction of the deep sequencing data to assess whether inferences are limited by sequencing depth or the underlying molecular diversity in the mutant library.

### The goal: inferring site-specific amino-acid preferences

The goal is to estimate the effects of mutations from changes in their counts after selection. Let *μ*_*r*,*x*_, *f*_*r*,*x*_, $f_{r,x}^{s1}$, and $f_{r,x}^{s2}$ denote the *true* frequencies at site *r* of all mutant characters *x*≠ wt(*r*) that would be observed for the four libraries in Figure 1 if we sampled at infinite depth in both the actual experiment and the sequencing. The definition of these frequencies for the wildtype character wt(*r*) depends on how the mutant library is constructed. If the mutant library is constructed so that there is a Poisson distribution of the number of mutations per gene (as is the case for error-prone PCR or the codon-mutagenesis in [9,11]), then *μ*_*r*,wt(*r*)_, *f*_*r*,wt(*r*)_, $f_{r,\operatorname {wt}\left (r\right)}^{s1}$, and $f_{r,\operatorname {wt}\left (r\right)}^{s2}$ are defined as for all other characters *x*, and denote the frequencies of wt(*r*) at site *r* that would be observed if sampling at infinite depth. The reason we can make this definition for libraries containing genes with Poisson-distributed numbers of mutations is that for any reasonable-length gene (*L*≫1), the marginal distribution of the number of mutations in a gene is virtually unchanged by the knowledge that there is a mutation at site *r*. On the other hand, if the mutant library is constructed so that there is exactly zero or one mutation per gene (as in [8,10,15]), then the marginal distribution of the total number of mutations in a gene is changed by the knowledge that there is a mutation at *r*. In this case, the wildtype-character frequencies *μ*_*r*,wt(*r*)_, *f*_*r*,wt(*r*)_, $f_{r,\operatorname {wt}\left (r\right)}^{s1}$, and $f_{r,\operatorname {wt}\left (r\right)}^{s2}$ are correctly defined as the frequency of unmutated genes in the library, and the counts $n_{r,\operatorname {wt}\left (r\right)}^{\text {pre}}$, etc. are defined as the number of reads at *r* attributable to unmutated genes. In this case, measurement of these counts requires sequencing with linkage as in [15,23,24]. The proper analysis of libraries containing only unmutated and singly mutated clones sequenced without linkage is beyond the scope of this paper.

If we knew the frequencies *μ*_*r*,*x*_, *f*_*r*,*x*_, $f_{r,x}^{s1}$, and $f_{r,x}^{s2}$, we could calculate parameters that reflect the effects of mutations. One parameter that characterizes the effect of mutating *r* from wt(*r*) to *x* for the experiment in Figure 1A is the *enrichment ratio*, which is the relative frequency of mutations to *x* after selection versus before selection:

(1) $$  \phi_{r,x} = \frac{f_{r,x} / f_{r,\operatorname{wt}\left(r\right)}}{\mu_{r,x} / \mu_{r,\operatorname{wt}\left(r\right)}}.  $$

Beneficial mutations have *ϕ*_*r*,*x*_>1, while deleterious ones have *ϕ*_*r*,*x*_<1. A related parameter is the *preference* *π*_*r*,*x*_ of *r* for *x*. At each site, the preferences are simply the enrichment ratios rescaled to sum to one:

(2) $$  \pi_{r,x} = \frac{\phi_{r,x}}{\sum_{y} \phi_{r,y}} = \frac{f_{r,x} / \mu_{r,x}}{\sum_{y} f_{r,y} / \mu_{r,y}},  $$

or equivalently

(3) $$  f_{r,x} = \frac{\pi_{r,x} \times \mu_{r,x}}{\sum_{y} \pi_{r,y} \times \mu_{r,y}},  $$

where *y* is summed over all character identities (all nucleotides, codons, or amino acids). The preferences can be intuitively visualized (Figure 1A) and interpreted as the equilibrium frequencies in substitution models for gene evolution [9,25] (after accounting for uneven mutational rates [26,27]).

### The challenge of statistical inference from finite counts

Equations 1 and 2 are in terms of the true frequencies *μ*_*r*,*x*_, *f*_*r*,*x*_, etc. But in practice, we only observe the counts in the finite sample of sequenced molecules. The computational challenge is to estimate the preferences (or enrichment ratios) from these counts.

The most naive approach is to simply substitute the counts for the frequencies, replacing Equation 1 with

(4) $$  \phi_{r,x} = \frac{\frac{n_{r,x}^{\text{post}} + \mathcal{P}}{n_{r,\operatorname{wt}\left(r\right)}^{\text{post}} + \mathcal{P}}}{\frac{n_{r,x}^{\text{pre}} + \mathcal{P}}{n_{r,\operatorname{wt}\left(r\right)}^{\text{pre}} + \mathcal{P}}}  $$

where  (often chosen to be one) is a pseudocount added to each count to avoid ratios of zero or infinity.

However, Equation 4 involves ratios of counts with values ∼10 to 100 – and as originally noted by Karl Pearson [28,29], ratios estimated from finite counts are statistically biased, with the bias increasing as the magnitude of the counts decrease. This bias can propagate into subsequent analyses, since many statistical tests assume symmetric errors. The problems caused by biases in uncorrected ratios have been noted even in applications such as isotope-ratio mass spectrometry [30] and fluorescent imaging [31], where the counts usually far exceed those in deep mutational scanning.

Taking ratios also abrogates our ability to use the magnitude of the counts to assess our certainty about conclusions. For instance, imagine that at a fixed depth, the counts of a mutation increase from a pre-selection value of 5 to a post-selection value of 10. While this doubling suggests that the mutation might be beneficial, the small counts make us somewhat uncertain of this conclusion. But if the counts increased from 20 to 40 we would be substantially more certain, and if they increased from 100 to 200 we would be quite sure. So only by an explicit statistical treatment of the counts can we fully leverage the data.

Here I describe a software package, dms_tools, that infers mutational effects in a Bayesian framework using a likelihood-based treatment of the counts. This software can be used to infer and visualize site-specific preferences from experiments like Figure 1A, and to infer and visualize differences in preferences under alternative selections from experiments like Figure 1B.

## Implementation and results

### Algorithm to infer site-specific preferences

dms_tools uses a Bayesian approach to infer site-specific preferences from experiments like those in Figure 1A. The algorithm calculates the likelihoods of the counts given the unknown preferences and mutation/error rates, placing plausible priors over these unknown parameters. The priors correspond to the assumption that all possible identities (e.g. amino acids) have equal preferences, and that the mutation and error rates for each site are equal to the overall average for the gene. MCMC is used to calculate the posterior probability of the preferences given the counts.

This algorithm is a slight modification of that in the *Methods* of [9]; here the algorithm is described anew to explain the implementation in dms_tools.

#### Optional controls to quantify error rates

Some sequencing reads that report a mutation may actually reflect an error introduced during sequencing or PCR rather than an actual mutation that experienced selection. Errors can be quantified by sequencing an unmutated gene, so that any counts at *r* of *x*≠ wt(*r*) for this control reflect errors. In some cases (e.g. sequencing an RNA virus where the post-selection libraries must be reverse-transcribed), error rates for the pre- and post-selection libraries may differ and so be described by different controls. Let $N_{r}^{\text {errpre}}$ and $N_{r}^{\text {errpost}}$ be the depth and $n_{r,x}^{\text {errpre}}$ and $n_{r,x}^{\text {errpost}}$ be the counts of *x* in the pre-selection and post-selection error controls, respectively. Define *ε*_*r*,*x*_ and *ρ*_*r*,*x*_ to be the true frequencies of errors at *r* from wt(*r*) to *x* in the pre- and post-selection controls, respectively.

#### Likelihoods of observing specific mutational counts

Define vectors of the counts and frequencies for all characters at each site *r*, i.e. $\mathbf {n_{r}^{\textbf {pre}}}= \left (\cdots, n_{r,x}^{\text {pre}}, \cdots \right)$, $\mathbf {n_{r}^{\textbf {post}}}= \left (\cdots, n_{r,x}^{\text {post}}, \cdots \right)$, ***μ***_***r***_=(⋯,*μ*_*r*,*x*_,⋯), ***f***_***r***_=(⋯,*f*_*r*,*x*_,⋯), etc. Also define ***π***_***r***_=(⋯,*π*_*r*,*x*_,⋯) of the preferences for each *r*, noting that Equation 3 implies $\boldsymbol {\mathbf {f_{r}}} = \frac {\boldsymbol {\mathbf {\mu _{r}}}\circ \boldsymbol {\mathbf {\pi _{r}}}}{\boldsymbol {\mathbf {\mu _{r}}}\cdot \boldsymbol {\mathbf {\pi _{r}}}}$ where ∘ is the Hadamard product.

The likelihoods of some specific set of counts are:

(5) $$ \begin{aligned} \Pr&\left(\mathbf{n_{r}^{\textbf{errpre}}} \mid N_{r}^{\text{errpre}}, \boldsymbol{\mathbf{\epsilon_{r}}}\right) = \\& \operatorname{Multi}\left(\mathbf{n_{r}^{\textbf{errpre}}}; N_{r}^{\text{errpre}}, \boldsymbol{\mathbf{\epsilon_{r}}}\right) \end{aligned}  $$

(6) $$ \begin{aligned} \Pr&\left(\mathbf{n_{r}^{\textbf{errpost}}} \mid N_{r}^{\text{errpost}}, \boldsymbol{\mathbf{\rho_{r}}}\right) = \\ & \operatorname{Multi}\left(\mathbf{n_{r}^{\textbf{errpost}}}; N_{r}^{\text{errpost}}, \boldsymbol{\mathbf{\rho_{r}}}\right) \end{aligned}  $$

(7) $$ \begin{aligned} \Pr&\left(\mathbf{n_{r}^{\textbf{pre}}}\mid N_{r}^{\text{pre}}, \boldsymbol{\mathbf{\mu_{r}}}, \boldsymbol{\mathbf{\epsilon_{r}}}\right) = \\ &\operatorname{Multi}\left(\mathbf{n_{r}^{\textbf{pre}}}; N_{r}^{\text{pre}}, \boldsymbol{\mathbf{\mu_{r}}}+ \boldsymbol{\mathbf{\epsilon_{r}}}- \boldsymbol{\mathbf{\delta_{r}}}\right) \end{aligned}  $$

(8) $$ \begin{aligned} \Pr&\left(\mathbf{n_{r}^{\textbf{post}}} \mid N_{r}^{\text{post}}, \boldsymbol{\mathbf{\mu_{r}}}, \boldsymbol{\mathbf{\pi_{r}}}, \boldsymbol{\mathbf{\rho_{r}}}\right) = \\ &\operatorname{Multi}\left(\mathbf{n_{r}^{\textbf{post}}}; N_{r}^{\text{post}}, \frac{\boldsymbol{\mathbf{\mu_{r}}}\circ \boldsymbol{\mathbf{\pi_{r}}}}{\boldsymbol{\mathbf{\mu_{r}}}\cdot \boldsymbol{\mathbf{\pi_{r}}}} + \boldsymbol{\mathbf{\rho_{r}}}- \boldsymbol{\mathbf{\delta_{r}}}\right) \end{aligned}  $$

where Multi is the multinomial distribution, ***δ***_***r***_=(⋯,*δ*_*x*,wt(*r*)_,⋯) is a vector with the element corresponding to wt(*r*) equal to one and all other elements zero (*δ*_*xy*_ is the Kronecker delta), and we have assumed that the probability that a site experiences both a mutation and an error is negligibly small.

#### Priors over the unknown parameters

We specify Dirichlet priors over the parameter vectors:

(9) $$\begin{array}{*{20}l} \Pr\left(\boldsymbol{\mathbf{\pi_{r}}}\right) = \operatorname{Dirichlet}\left(\boldsymbol{\mathbf{\pi_{r}}}; \alpha_{\pi} \times \mathbf{1}\right) \end{array} $$

(10) $$\begin{array}{*{20}l}  \Pr\left(\boldsymbol{\mathbf{\mu_{r}}}\right) = \operatorname{Dirichlet}\left(\boldsymbol{\mathbf{\mu_{r}}}; \alpha_{\mu} \times \mathcal{N}_{x} \times \boldsymbol{\mathbf{a_{r,\mu}}} \right) \end{array} $$

(11) $$\begin{array}{*{20}l} \Pr\left(\boldsymbol{\mathbf{\epsilon_{r}}}\right) = \operatorname{Dirichlet}\left(\boldsymbol{\mathbf{\epsilon_{r}}}; \alpha_{\epsilon} \times \mathcal{N}_{x} \times \boldsymbol{\mathbf{a_{r,\epsilon}}} \right) \end{array} $$

(12) $$\begin{array}{*{20}l} \Pr\left(\boldsymbol{\mathbf{\rho_{r}}}\right) = \operatorname{Dirichlet}\left(\boldsymbol{\mathbf{\rho_{r}}}; \alpha_{\rho} \times \mathcal{N}_{x} \times \boldsymbol{\mathbf{a_{r,\rho}}}\right) \end{array} $$

where **1** is a vector of ones, $\mathcal {N}_{x}$ is the number of characters (64 for codons, 20 for amino acids, 4 for nucleotides), the *α*’s are scalar concentration parameters >0 (by default dms_tools sets the *α*’s to one). For codons, the error rate depends on the number of nucleotides being changed. The average error rates $\overline {\epsilon _{m}}$ and $\overline {\rho _{m}}$ for codon mutations with *m* nucleotide changes are estimated as

(13) $$\begin{array}{*{20}l} \overline{\epsilon_{m}} = \frac{1}{L}\sum\limits_{r} \frac{1}{N_{r}^{\text{errpre}}}\sum\limits_{x} n_{r,x}^{\text{errpre}}\times \delta_{m,D_{x,\operatorname{wt}\left(r\right)}}  \end{array} $$

(14) $$\begin{array}{*{20}l} \overline{\rho_{m}} = \frac{1}{L}\sum\limits_{r} \frac{1}{N_{r}^{\text{errpost}}}\sum\limits_{x} n_{r,x}^{\text{errpost}}\times \delta_{m,D_{x,\operatorname{wt}\left(r\right)}}  \end{array} $$

where *D*_*x*,wt(*r*)_ is the number of nucleotide differences between *x* and wt(*r*). Given these definitions,

(15) $$\begin{array}{*{20}l} \boldsymbol{\mathbf{a_{r,\epsilon}}} = \left(\cdots, \sum\limits_{m} \frac{\overline{\epsilon}_{m}}{\mathcal{C}_{m}} \times \delta_{m,D_{x,\operatorname{wt}\left(r\right)}},\cdots\right)  \end{array} $$

(16) $$\begin{array}{*{20}l} \boldsymbol{\mathbf{a_{r,\rho}}} = \left(\cdots, \sum\limits_{m} \frac{\overline{\rho}_{m}}{\mathcal{C}_{m}} \times \delta_{m,D_{x,\operatorname{wt}\left(r\right)}}, \cdots\right)  \end{array} $$

where $\mathcal {C}_{m}$ is the number of mutant characters with *m* changes relative to wildtype (for nucleotides $\mathcal {C}_{0} = 1$ and $\mathcal {C}_{1} = 3$; for codons $\mathcal {C}_{0} = 1$, $\mathcal {C}_{1} = 9$, $\mathcal {C}_{2} = \mathcal {C}_{3} = 27$).

Our prior assumption is that the mutagenesis introduces all mutant characters at equal frequency (this assumption is only plausible for codons if the mutagenesis is at the codon level as in [8-10,15-17]; if mutations are made at the nucleotide level such as by error-prone PCR then characters should be defined as nucleotides). The average per-site mutagenesis rate is estimated as

(17) $$ \overline{\mu} = \left(\frac{1}{L}\sum\limits_{r} \frac{1}{N_{r}^{\text{pre}}}\sum\limits_{x\ne \operatorname{wt}\left(r\right)} n_{r,x}^{\text{pre}}\right) - \sum\limits_{m \ge 1} \overline{\epsilon_{m}},  $$

so that

(18) $$ \boldsymbol{\mathbf{a_{r,\mu}}} = \left(\cdots, \frac{\overline{\mu}}{\mathcal{N}_{x} - 1} + \delta_{x,\operatorname{wt}\left(r\right)} \times \left[1 - \overline{\mu}\right],\cdots\right).  $$

#### Character types: nucleotides, amino acids, or codons

dms_tools allows four possibilities for the type of character for the counts and preferences. The first three possibilities are simple: the counts and preferences can both be for any of nucleotides, amino acids, or codons.

The fourth possibility is that the counts are for codons, but the preferences for amino acids. In this case, define a function mapping codons to amino acids,

(19) $$ \mathbf{A}\left(\mathbf{w}\right) = \left(\cdots, \sum\limits_{x} \delta_{a,\mathcal{A}\left(x\right)} \times w_{x}, \cdots\right)  $$

where **w** is a 64-element vector of codons *x*, **A**(**w**) is a 20- or 21-element (depending on the treatment of stop codons) vector of amino acids *a*, and $\mathcal {A}\left (x\right)$ is the amino acid encoded by *x*. The prior over the preferences ***π***_***r***_ is still a symmetric Dirichlet (now only of length 20 or 21), but the priors for ***μ***_***r***_, ***ε***_***r***_, and ***ρ***_***r***_ are now Dirichlets parameterized by **A**(***a***_***r,μ***_), **A**(***a***_***r,ε***_) and **A**(***a***_***r,ρ***_) rather than ***a***_***r,μ***_, ***a***_***r,ε***_, and ***a***_***r,ρ***_. The likelihoods are computed in terms of these transformed vectors after similarly transforming the counts to $\mathbf {A}\left (\mathbf {n_{r}^{\textbf {pre}}}\right)$, $\mathbf {A}\left (\mathbf {n_{r}^{\textbf {post}}}\right)$, $\mathbf {A}\left (\mathbf {n_{r}^{\textbf {errpre}}}\right)$, and $\mathbf {A}\left (\mathbf {n_{r}^{\textbf {errpost}}}\right)$.

#### Implementation

The program dms_inferprefs in the dms_tools package infers the preferences by using pystan [32] to perform MCMC over the posterior defined by the product of the likelihoods and priors in Equations 5, 6, 7, 8, 9, 10, 11, and 12. The program runs four chains from different initial values, and checks for convergence by ensuring that the Gelman-Rubin statistic $\hat {R}$ [33] is <1.1 and the effective sample size is >100; the number of MCMC iterations is increased until convergence is achieved. The program dms_logoplot in the dms_tools package visualizes the posterior mean preferences via an extension to weblogo [34]. The program dms_merge can be used to average preferences inferred from different experimental replicates that have individually been analyzed by dms_inferprefs, and the program dms_correlate can be used to compute the correlations among inferences from different replicates.

### Inferring preferences with dms_tools

#### Application to actual datasets

**Figure 2:**
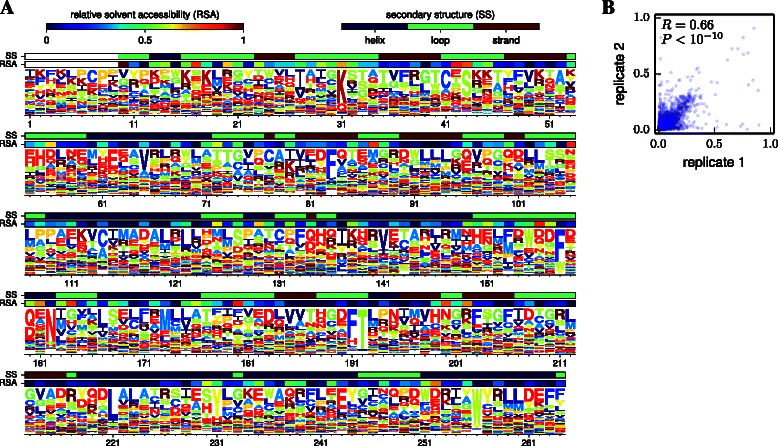
Site-specific preferences from deep mutational scanning of a Tn5 transposon. Melnikov *et al.* [10] performed deep mutational scanning on a Tn5 transposon using kanamycin selection, and reported the counts of amino-acid mutations for two biological replicates of the experiment. Here I have used dms_tools to infer the preferences. **(A)** Visualization of the preferences averaged across the two replicates. **(B)** Correlation between the preferences inferred from each of the two replicates. Given files containing the mutation counts, the plots can be generated as logoplot.pdf and corr.pdf with the following commands:.

**Figure 3:**
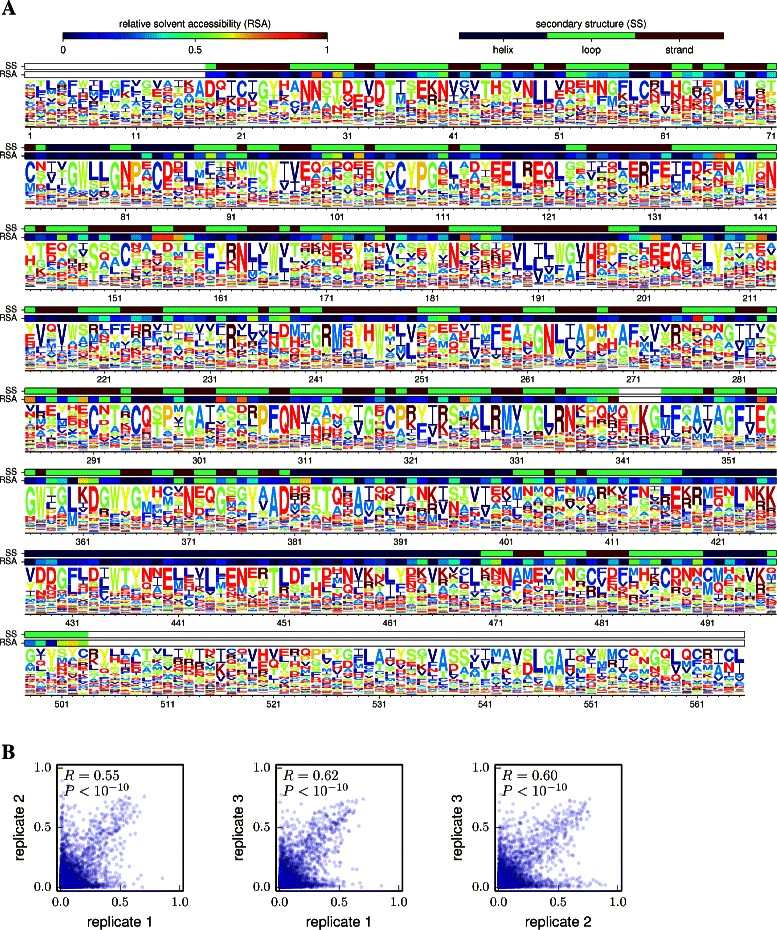
Site-specific preferences from deep mutational scanning of influenza hemagglutinin. Thyagarajan and Bloom [11] performed deep mutational scanning on influenza hemagglutinin, and reported the counts of codon mutations for three biological replicates of the experiment. Here I have used dms_tools to infer the preferences. **(A)** Visualization of the preferences averaged across the three replicates. **(B)** Correlations between the preferences from each pair of replicates. Given files containing the mutation counts, the plots can be generated as logoplot.pdf, corr_1_2.pdf, corr_1_3.pdf, and corr_2_3.pdf with the following commands:.

Figures 2 and 3 illustrate application of dms_tools to two existing datasets [10,11]. The programs require as input only simple text files listing the counts of each character identity at each site. As the figures show, the dms_inferprefs and dms_logoplot programs can process these input files to infer and visualize the preferences with a few simple commands. Error controls can be included when available (they are not for Figure 2, but are for Figure 3). The runtime for the MCMC depends on the gene length and character type (codons are slowest, nucleotides fastest) – but if the inference is parallelized across multiple CPUs (using the –ncpus option of dms_inferprefs), the inference should take no more than a few hours. As shown in Figures 2 and 3, the visualizations can overlay information about protein structure onto the preferences.

Figures 2 and 3 also illustrate use of dms_correlate to assess the correlation between preferences inferred from different biological replicates [35] of the experiment. The inclusion and analysis of such replicates is the only sure way to fully assess the sources of noise associated with deep mutational scanning.

#### Testing on simulated data

**Figure 4:**
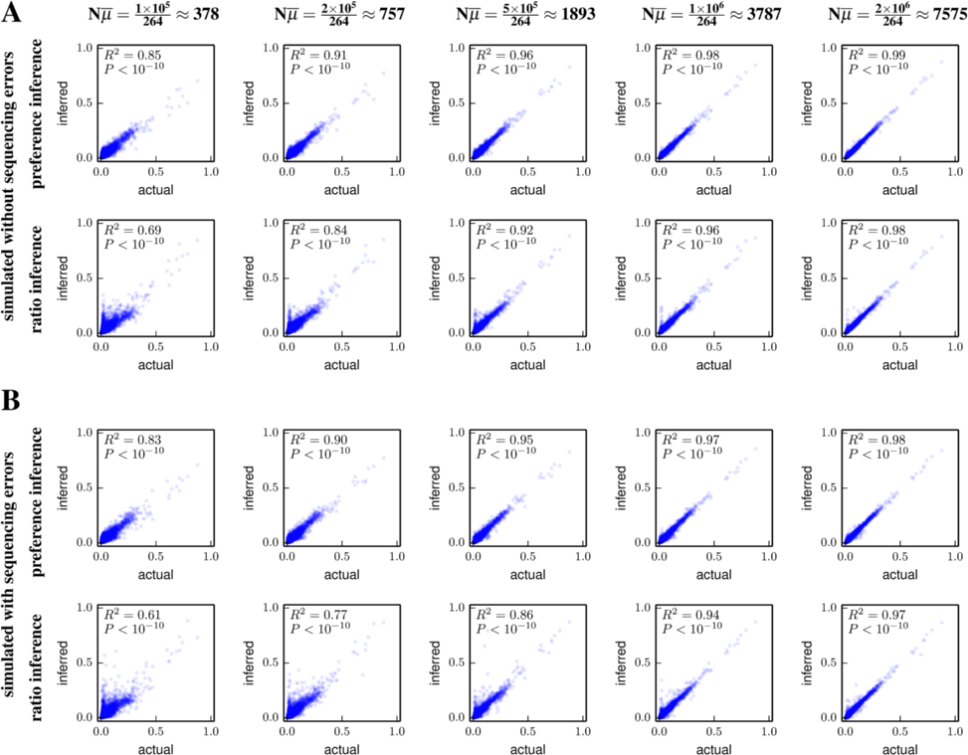
Accuracy of preference inference on simulated data. Deep mutational scanning counts were simulated using the preferences in Figure 2A and realistic mutation and error rates that were uneven across sites and characters as in actual experiments. The simulations were done **(A)** without or **(B)** with sequencing errors quantified by control libraries. Plots show the correlation between the actual and inferred preferences as a function of the product of the sequencing depth *N* and the average per-site mutation rate $\overline {\mu }$; real experiments typically have $N\overline {\mu } \sim 1000$ to 2000 depending on the sequencing depth and gene length. Preferences are inferred using the full algorithm in dms_tools (top panels) or by simply calculating ratios of counts (bottom panels) using Equation 4 and its logical extension to include errors, both with a pseudocount of one. The dms_tools inferences are more accurate than the simple ratio estimation, with both methods converging to the actual values with increasing $N\overline {\mu }$. Given files with the mutation counts, the plots in this figure can be generated as prefs_corr.pdf and ratio_corr.pdf with commands such as:.

To test the accuracy of preference-inference by dms_tools, I simulated deep mutational scanning counts using the preferences in Figure 2, both with and without errors quantified by appropriate controls. Importantly, the error and mutation rates for these simulations were *not* uniform across sites and characters, but were simulated to have a level of unevenness comparable to that observed in real experiments. I then used dms_tools to infer preferences from the simulated data, and also made similar inferences using simple ratio estimation (Equation 4). Figure 4 shows the inferred preferences versus the actual values used to simulate the data. For simulations with mutation counts (quantified by the product $N\overline {\mu }$ of the depth and average per-site mutation rate) ∼1000 to 2000, the inferences are quite accurate. Inferences made by dms_tools are always more accurate than those obtained by simply taking ratios of mutation counts.

#### Is the Bayesian inference worthwhile?

The foregoing sections explain why the Bayesian inference of preferences implemented in dms_tools is conceptually preferable to estimating mutational effects via direct ratio estimation using Equation 4. However, do the practical benefits of this Bayesian inference justify its increased complexity? The simulations in the previous section show that the Bayesian inference is more accurate, but in the absence of background errors (Figure 4A) the magnitude of the improvement becomes negligible once the mutation counts per site $N \overline {\mu }$ start to exceed ∼10^3^. When there is a need to correct for background errors (Figure 4B), meaningful benefits of the Bayesian inference over enrichment ratios extend to somewhat higher sequencing depths. Overall, it appears that Bayesian inference will always perform as well or better than ratio estimation, but that the tangible benefit becomes negligible at high sequencing depth. In that case, the user will have to decide if the increased computational runtime and complexity of the Bayesian inference is worth a small improvement in accuracy. Simpler ratio estimation can be performed using the –ratio_estimation option of dms_inferprefs or using an alternative program such as Enrich [19]. When applying ratio estimation to data where some mutations have low counts, it is important to include pseudocounts (denoted by  in Equation 4) as a form of regularization to avoid estimating excessively high or low preferences at sites with limited counts.

### Algorithm to infer differential preferences

As shown in Figure 1B, a useful extension to the experiment in Figure 1A is to subject the functional variants to two different selection pressures to identify mutations favored by one pressure versus the other. While this experiment could in principle by analyzed by simply comparing the initial unselected mutants to the final variants after the two alternative selections, this approach is non-ideal. In experiments like Figure 1A, many mutations are enriched or depleted to some extent by selection, since a large fraction of random mutations affect protein function [36-40]. Therefore, the assumption that all mutations are equally tolerated (i.e. the preferences for a site are all equal, or the enrichment ratios are all one) is not a plausible null hypothesis for Figure 1A. For this reason, dms_tools simply infers the preferences given a uniform Dirichlet prior rather than trying to pinpoint some subset of sites with unequal preferences.

But in Figure 1B, the assumption that most mutations will be similarly selected is a plausible null hypothesis, since we expect alternative selections to have markedly different effects on only a small subset of mutations (typically, major constraints related to protein folding and stability will be conserved across different selections on the same protein). Therefore, dms_tools uses a different algorithm to infer the differential preferences under the two selections. This algorithm combines a prior that mildly favors differential preferences of zero with a likelihood-based analysis of the mutation counts to estimate posterior probabilities over the differential preferences.

#### Definition of the differential preferences

Given an experiment like Figure 1B, let $f_{r,x}^{\text {start}}$ be the true frequency of character *x* at site *r* in the starting library (equivalent to the frequency $f_{r,x}^{\text {post}}$ in the figure), and let $f_{r,x}^{s1}$ and $f_{r,x}^{s2}$ be the frequencies after selections *s*1 and *s*2, respectively. The differential preference *Δ* *π*_*r*,*x*_ for *x* at *r* in *s*2 versus *s*1 is defined by:

(20) $$ \begin{aligned} f_{r,x}^{s1} = \frac{f_{r,x}^{\text{start}} \times \pi_{r,x}^{s1}}{\sum_{y} f_{r,y}^{\text{start}} \times \pi_{r,y}^{s1}} \end{aligned}  $$

(21) $$ \begin{aligned} f_{r,x}^{s2} = \frac{f_{r,x}^{\text{start}} \times \left(\pi_{r,x}^{s1} + \Delta\pi_{r,x}\right)}{\sum_{y} f_{r,y}^{\text{start}} \times \left(\pi_{r,y}^{s1} + \Delta\pi_{r,y}\right)} \end{aligned}  $$

where $\pi _{r,x}^{s1}$ is the “control preference” and is treated as a nuisance parameter, and we have the constraints

(22) $$ \begin{aligned} 0 = \sum\limits_{x} \Delta\pi_{r,x} \end{aligned}  $$

(23) $$ \begin{aligned} 0 \le \pi_{r,x}^{s1} + \Delta\pi_{r,x} \le 1. \end{aligned}  $$

**Figure 5:**
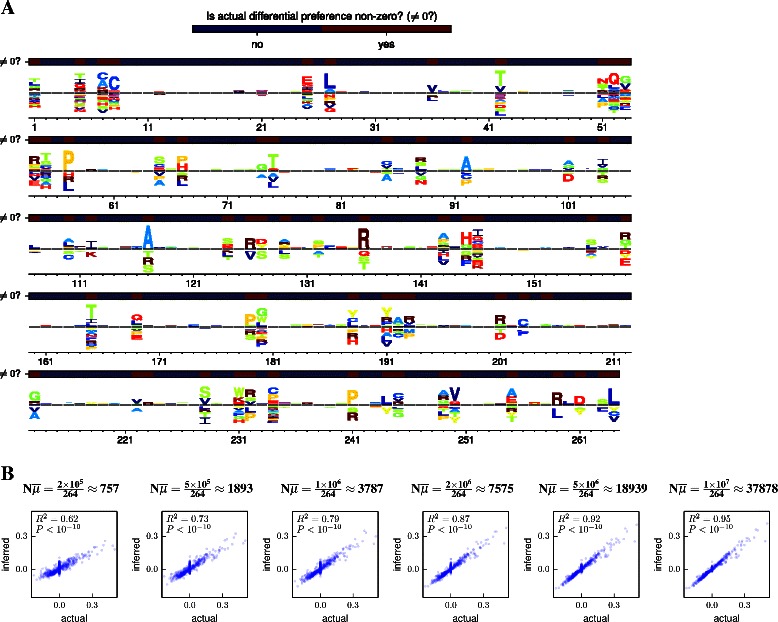
Inference of differential preferences on simulated data. To illustrate and test the inference of differential preferences, the experiment in Figure 1B was simulated at the codon level starting with the post-selection library that yielded the preferences in Figure 2. In the simulations, 20% of sites had different preferences between the control and alternative selection. **(A)**, dms_tools was used to infer the differential preferences from the data simulated at *N*=10^7^, and the resulting inferences were visualized. The overlay bars indicate which sites had non-zero differential preferences in the simulation. **(B)** The correlations between the inferred and actual differential preferences as a function of $N\overline {\mu }$ show that the inferred values converge to the true ones. Given files with the mutation counts, the plots in this figure can be generated as logoplot.pdf and corr.pdf with the following commands:.

If there is no difference in the effect of *x* at *r* between selections *s*1 and *s*2, then *Δ* *π*_*r*,*x*_=0. If *x* at *r* is more preferred by *s*2 than *s*1, then *Δ* *π*_*r*,*x*_>0; conversely if *x* at *r* is more preferred by *s*1 than *s*2, then *Δ* *π*_*r*,*x*_<0 (see Figure 5A).

#### Likelihoods of observing specific mutational counts

Define vectors of the counts as $\mathbf {n_{r}^{start}}= \left (\cdots, n_{r,x}^{\text {start}}, \cdots \right)$ for the post-selection functional variants that are subjected to the further selections, and as $\mathbf {n_{r}^{s1}}= \left (\cdots, n_{r,x}^{s1}, \cdots \right)$ and $\mathbf {n_{r}^{s2}}= \left (\cdots, n_{r,x}^{s2}, \cdots \right)$ for selections *s*1 and *s*2. We again allow an error control, but now assume that the same control applies to all three libraries (since they are all sequenced after a selection), and define the counts for this control as $\mathbf {n_{r}^{\textbf {err}}}= \left (\cdots, n_{r,x}^{\text {err}}, \cdots \right)$; the true error frequencies are denoted by *ξ*_*r*,*x*_. Define vectors of the frequencies, errors, control preferences, and differential preferences: $\mathbf {f_{r}^{\,\textbf {start}}}= \left (\cdots, f_{r,x}^{\text {start}}, \cdots \right)$, $\mathbf {f_{r}^{\,s1}}= \left (\cdots, f_{r,x}^{s1}, \cdots \right)$, $\mathbf {f_{r}^{\,s2}}= \left (\cdots, f_{r,x}^{s2}, \cdots \right)$, ***ξ***_***r***_=(⋯,*ξ*_*r*,*x*_,⋯), $\boldsymbol {\mathbf {\pi ^{s1}_{r}}}= \left (\cdots, \pi ^{s1}_{r,x}, \cdots \right)$, and ***Δπ***_***r***_=(⋯,*Δ* *π*_*r*,*x*_,⋯). Equations 20 and 21 imply $\mathbf {f_{r}^{\,s1}}= \frac {\mathbf {f_{r}^{\,\textbf {start}}}\circ \mathbf {\pi _{r}^{s1}}}{\mathbf {f_{r}^{\,\textbf {start}}}\cdot \mathbf {\pi _{r}^{s1}}}$ and $\mathbf {f_{r}^{\,s2}} = \frac {\mathbf {f_{r}^{\textbf {start}}}\circ \left (\mathbf {\pi _{r}^{s1}} + \mathbf {\Delta \pi _{r}}\right)}{\mathbf {f_{r}^{\,\textbf {start}}}\cdot \left (\mathbf {\pi _{r}^{s1}} + \mathbf {\Delta \pi _{r}}\right)}$.

The likelihoods of the counts will be multinomially distributed around the “true” frequencies, so

(24) $$ \begin{aligned}  \Pr\left(\mathbf{n_{r}^{\textbf{err}}}\mid N_{r}^{\text{err}}, \boldsymbol{\mathbf{\xi_{r}}}\right) = \operatorname{Multi}\left(\mathbf{n_{r}^{\textbf{err}}}; N_{r}^{\text{err}}, \boldsymbol{\mathbf{\xi_{r}}}\right) \end{aligned}  $$

(25) $$ \begin{aligned}  \Pr&\left(\mathbf{n_{r}^{\textbf{start}}}\mid N_{r}^{\text{start}}, \boldsymbol{\mathbf{f_{r}^{\textbf{\,start}}}}, \boldsymbol{\mathbf{\xi_{r}}}\right) = \\ & \operatorname{Multi}\left(\mathbf{n_{r}^{\textbf{start}}}; N_{r}^{\text{start}}, \boldsymbol{\mathbf{f_{r}^{\textbf{\,start}}}} + \boldsymbol{\mathbf{\xi_{r}}}- \boldsymbol{\mathbf{\delta_{r}}}\right) \end{aligned}  $$

(26) $$ \begin{aligned}  \Pr&\left(\mathbf{n_{r}^{s1}}\mid N_{r}^{s1}, \boldsymbol{\mathbf{f_{r}^{\textbf{\,start}}}}, \boldsymbol{\mathbf{\pi_{r}^{s1}}}, \boldsymbol{\mathbf{\xi_{r}}}\right) = \\ & \operatorname{Multi}\left(\mathbf{n_{r}^{s1}}; N_{r}^{s1}, \frac{\boldsymbol{\mathbf{f_{r}^{\textbf{\,start}}}}\circ \boldsymbol{\mathbf{\pi_{r}^{s1}}}}{\boldsymbol{\mathbf{f_{r}^{\textbf{\,start}}}}\cdot \boldsymbol{\mathbf{\pi_{r}^{s1}}}} + \boldsymbol{\mathbf{\xi_{r}}}- \boldsymbol{\mathbf{\delta_{r}}}\right) \end{aligned}  $$

(27) $${} \begin{aligned}  \Pr&\left(\mathbf{n_{r}^{s2}}\mid N_{r}^{s2}, \boldsymbol{\mathbf{f_{r}^{\textbf{\,start}}}}, \boldsymbol{\mathbf{\pi_{r}^{s1}}}, \boldsymbol{\mathbf{\Delta\pi_{r}}}, \boldsymbol{\mathbf{\xi_{r}}}\right) = \\ & \operatorname{Multi}\left(\mathbf{n_{r}^{s2}}; N_{r}^{s2}, \frac{\boldsymbol{\mathbf{f_{r}^{\textbf{\,start}}}}\circ \left(\boldsymbol{\mathbf{\Delta\pi_{r}}} + \boldsymbol{\mathbf{\pi_{r}^{s1}}}\right)}{\boldsymbol{\mathbf{f_{r}^{\textbf{\,start}}}}\cdot \left(\boldsymbol{\mathbf{\Delta\pi_{r}}} + \boldsymbol{\mathbf{\pi_{r}^{s1}}}\right)} + \boldsymbol{\mathbf{\xi_{r}}}- \boldsymbol{\mathbf{\delta_{r}}}\right) \end{aligned}  $$

where we have assumed that the probability that a site experiences a mutation and an error in the same molecule is negligibly small.

#### Priors over the unknown parameters

We specify Dirichlet priors over the parameter vectors:

(28) $$\begin{array}{@{}rcl@{}} \Pr\left(\boldsymbol{\mathbf{\pi_{r}^{s1}}}\right) = \operatorname{Dirichlet}\left(\boldsymbol{\mathbf{\pi_{r}^{s1}}}; \alpha_{\pi^{s1}} \times \mathbf{1}\right) \end{array} $$

(29) $$\begin{array}{@{}rcl@{}}  \Pr\left(\boldsymbol{\mathbf{\xi_{r}}}\right) = \operatorname{Dirichlet}\left(\boldsymbol{\mathbf{\xi_{r}}}; \alpha_{\xi} \times \mathcal{N}_{x} \times \boldsymbol{\mathbf{a_{r,\xi}}}\right) \end{array} $$

$$\begin{array}{@{}rcl@{}}  \Pr\left(\boldsymbol{\mathbf{f_{r}^{\,\textbf{start}}}}\right) = \notag \end{array} $$

(30) $$\begin{array}{@{}rcl@{}} \quad\;\operatorname{Dirichlet}\left(\boldsymbol{\mathbf{f_{r}^{\,\textbf{start}}}}; \alpha_{\text{start}} \times \mathcal{N}_{x} \times \boldsymbol{\mathbf{a_{r,\textbf{start}}}}\right)~~~~ \end{array} $$

(31) $$ \begin{aligned} \Pr&\left(\boldsymbol{\mathbf{\Delta\pi_{r}}} \mid \boldsymbol{\mathbf{\pi_{r}^{s1}}}\right) = \\ & \operatorname{Dirichlet}\left(\boldsymbol{\mathbf{\Delta\pi_{r}}}; \alpha_{\Delta\pi} \times \mathcal{N}_{x} \times \boldsymbol{\mathbf{\pi_{r}^{s1}}}\right) - \boldsymbol{\mathbf{\pi_{r}^{s1}}} \end{aligned}  $$

where dms_tools by default sets all the scalar concentration parameters (*α*’s) to one except *α*_*Δπ*_, which is set to two corresponding to a weak expectation that the *Δ* *π* values are close to zero. The average error rate $\overline {\xi _{m}}$ for mutations with *m* nucleotide changes is

(32) $$ \overline{\xi_{m}} = \frac{1}{L}\sum\limits_{r} \frac{1}{N_{r}^{\text{err}}}\sum\limits_{x} n_{r,x}^{\text{err}}\times \delta_{m,D_{x,\operatorname{wt}\left(r\right)}},  $$

and so

(33) $$ \boldsymbol{\mathbf{a_{r,\xi}}} = \left(\cdots, \sum\limits_{m} \frac{\overline{\xi}_{m}}{\mathcal{C}_{m}} \times \delta_{m,D_{x,\operatorname{wt}\left(r\right)}},\cdots\right).  $$

Our prior assumption is that all mutations are at equal frequency in the starting library (this assumption is unlikely to be true if the starting library has already been subjected to some selection, but we lack a rationale for a more informative prior). The average mutation frequency in the starting library is

(34) $$ \overline{f^{\text{start}}} = \left(\frac{1}{L}\sum\limits_{r} \frac{1}{N_{r}^{\text{start}}}\sum\limits_{x\ne \operatorname{wt}\left(r\right)} n_{r,x}^{\text{start}}\right) - \sum\limits_{m \ge 1} \overline{\xi_{m}},  $$

and so

(35) $$ \boldsymbol{\mathbf{a_{r,\textbf{start}}}}= \left(\cdots, \frac{\overline{f^{\text{start}}}}{\mathcal{N}_{x} - 1} + \delta_{x,\operatorname{wt}\left(r\right)} \times \left[1 - \overline{f^{\text{start}}}\right],\cdots\right).  $$

#### Implementation

The program dms_inferdiffprefs in the dms_ tools package infers the differential preferences by performing MCMC over the posterior defined by the product of the likelihoods and priors in Equations 24, 25, 26, 27, 28, 29, 30, and 31. The MCMC is performed as described for the preferences, and characters can again be any of nucleotides, amino acids, or codons. The program dms_logoplot visualizes the posterior mean differential preferences via an extension to weblogo [34]. In addition, dms_inferdiffprefs creates text files that give the posterior probability that *Δ* *π*_*r*,*x*_>0 or <0. These posterior probabilities are *not* corrected to account for the fact that multiple sites are typically being examined, although by default the inferences are made using the regularizing prior in Equation 31.

### Inferring differential preference with dms_tools

**Figure 6:**
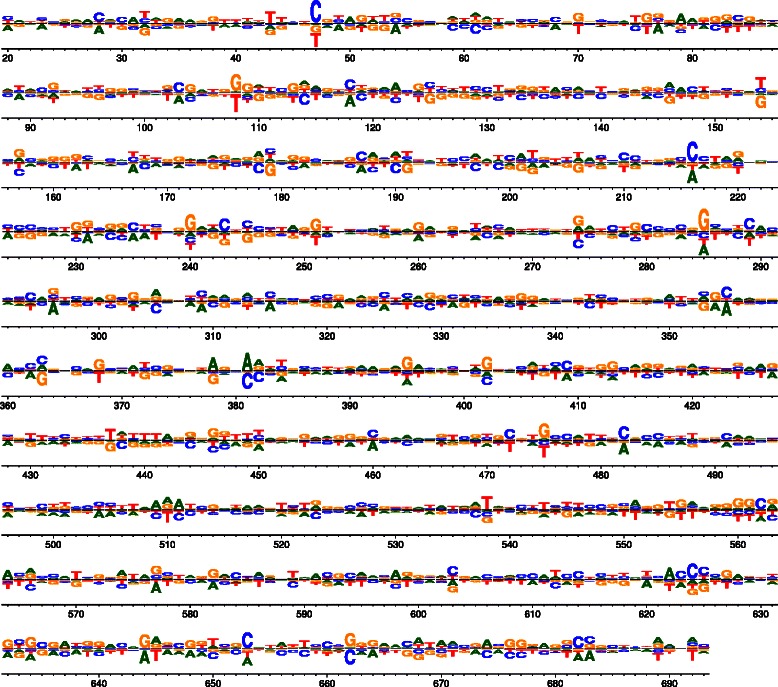
Differential preferences following selection of influenza NS1 in the presence or absence of interferon. Wu *et al.* [13] generated libraries of influenza viruses carrying nucleotide mutations in the NS segment. They passaged these viruses in the presence or absence of interferon pre-treatment. Here, dms_tools was used to analyze and visualize the data to identify sites where different nucleotides are preferred in the presence versus the absence of interferon. Because the mutations were made at the nucleotide level, the data must also be analyzed at that level (unlike in Figures 2, 3, and 5, where codon mutagenesis means that the data can be analyzed at the amino-acid level). The plot can be generated as logoplot.pdf with the following commands:.

To test the accuracy of differential preference inference by dms_tools, I simulated an experiment like that in Figure 1B with the starting counts based on Melnikov *et al.*’s actual deep mutational scanning data of a Tn5 transposon [10]. As shown by Figure 5, dms_inferdiffprefs accurately infers the differential preferences at typical experimental depths. The results are easily visualized with dms_logoplot. To provide a second illustration of differential preferences, Figure 6 shows an analysis of the data obtained by Wu *et al.* when they performed an experiment like that in Figure 1B on nucleotide mutants of the influenza NS gene in the presence or absence of interferon treatment.

## Conclusions

dms_tools is a freely available software package that uses a statistically principled approach to analyze deep mutational scanning data. This paper shows that dms_tools accurately infers preferences and differential preferences from data simulated under realistic parameters. As the figures illustrate, dms_tools can also be applied to actual data with a few simple commands. The intuitive visualizations created by dms_tools assist in interpreting the results. As deep mutational scanning continues to proliferate as an experimental technique [1], dms_tools can be applied to analyze the data for purposes such as guiding protein engineering [3,10], understanding sequence-structure-function relationships [4,5,7,14,21], informing the development of better evolutionary models for sequence analysis [9,25], and probing the biology of viruses and cells [6,8,11-13,18].

## Availability and requirements

- **Project name:** dms_tools
- **Project home page:**
  - Documentation and installation instructions: http://jbloom.github.io/dms_tools/
  - Source code: https://github.com/jbloom/dms_tools
- **Operating system(s):** Linux
- **Programming language:** Python
- **Other requirements:** pystan, weblogo
- **License:** GNU GPLv3
- **Restrictions to use by non-academics:** None

## Data and code for figures in this paper

The data and computer code used to generate the figures are in version 1.01 of the dms_tools source code (which is tagged on Github at https://github.com/jbloom/dms_tools/tree/1.0.1) in the examples subdirectory. The LaTex source for this paper is in the paper subdirectory.
